# Diversity-oriented synthesis and activity evaluation of substituted bicyclic lactams as anti-malarial against *Plasmodium falciparum*

**DOI:** 10.1186/1475-2875-13-467

**Published:** 2014-11-28

**Authors:** Vijeta Sharma, Shalini Agarwal, Sanjay M Madurkar, Gaurav Datta, Poonam Dangi, Ramu Dandugudumula, Subhabrata Sen, Shailja Singh

**Affiliations:** Shiv Nadar University, Gautam Budh Nagar, UP 203207 India; Malaria Group, International Centre for Genetic Engineering and Biotechnology (ICGEB), New Delhi, 110067 India; Department of Chemistry, Mahatma Gandhi University, 13th Mile, G S Road, Khanapara, Ri-Bhoi, Meghalaya 793101 India

**Keywords:** Malaria, DOS, Bicyclic lactams, Anti-malarial activity, *Plasmodium falciparum*

## Abstract

**Background:**

Malaria remains the world’s most important devastating parasitic disease. Of the five species of *Plasmodium* known to infect and cause human malaria, *Plasmodium falciparum* is the most virulent and responsible for majority of the deaths caused by this disease. Mainstream drug therapy targets the asexual blood stage of the malaria parasite, as the disease symptoms are mainly associated with this stage. The prevalence of malaria parasite strains resistance to existing anti-malarial drugs has made the control of malaria even more challenging and hence the development of a new class of drugs is inevitable.

**Methods:**

Screening against different drug resistant and sensitive strains of *P. falciparum* was performed for few bicyclic lactam-based motifs, exhibiting a broad spectrum of activity with low toxicity generated *via* a focussed library obtained from diversity oriented synthesis (DOS). The synthesis and screening was followed by an *in vitro* assessment of the possible cytotoxic effect of this class of compounds on malaria parasite.

**Results:**

The central scaffold a chiral bicyclic lactam (**A**) and (**A’**) which were synthesized from (**R**)-phenylalaninol, levulinic acid and 3-(2-nitrophenyl) levulinic acid respectively. The DOS library was generated from **A** and from **A’**, by either direct substitution with *o*-nitrobenzylbromide at the carbon α- to the amide functionality or by conversion to fused pyrroloquinolines. Upon screening this diverse library for their anti-malarial activity, a dinitro/diamine substituted bicyclic lactam was found to demonstrate exceptional activity of >85% inhibition at 50 μM concentration across different strains of *P. falciparum* with no toxicity against mammalian cells. Also, loss of mitochondrial membrane potential, mitochondrial functionality and apoptosis was observed in parasite treated with diamine-substituted bicyclic lactams.

**Conclusions:**

This study unveils a DOS-mediated exploration of small molecules with novel structural motifs that culminates in identifying a potential lead molecule against malaria. *In vitro* investigations further reveal their cytocidal effect on malaria parasite growth. It is not the first time that DOS has been used as a strategy to identify therapeutic leads against malaria, but this study establishes the direct implications of DOS in scouting novel motifs with anti-malarial activity.

**Electronic supplementary material:**

The online version of this article (doi:10.1186/1475-2875-13-467) contains supplementary material, which is available to authorized users.

## Background

Malaria, a leading cause of morbidity and mortality, is still the world’s most important parasitic disease [1]. Globally, an estimated 3.4 billion people are at risk of malaria. Out of all known species of malaria parasite, *Plasmodium falciparum* causes the most virulent form of malaria and is a leading infectious cause of morbidity and mortality [2]. Of the nearly 1,400 drugs registered worldwide in the last quarter of the 20th Century, only four were anti-malarials [3]. No promising and efficient anti-malarial vaccine is available despite various vaccines being produced and tested [4, 5]. Adding to the woes are the prevalent *P. falciparum* strains resistant to the anti-malarial drugs, such as chloroquine and artemisinin [6, 7]. Despite this resistance, combination-based therapy can continue to cure patients provided novel drugs are introduced and included in the combination [8]. Therefore, prompt endeavours are prerequisite to develop a new class of drugs as chemotherapeutic anti-malarials.

Diversity oriented synthesis (DOS) is one of the leading approaches in present day drug discovery to identify new class of compounds. It is considered one of the most useful techniques, to provide distinct molecular scaffolds that occupy different regions of chemical space [9]. It utilizes a combination of building block-, appendage-, stereochemical-, and skeletal-diversity to access structurally disparate compounds [10]. Among these, skeletal diversity, that deals with the scaffold architecture of the small molecules, is the most important to attain.

Herein, chiral bicyclic lactams (**A** and **A**’) (the central scaffolds of DOS-based strategy) were synthesized from (**R**)-phenylalaninol/levulinic acid and 3-(2-nitro)levulinic acid. The diversification strategies involved: (a) enolate-mediated substitution with *o*-nitrobenzylbromide at the carbon *α* –to the amide functionality (from **A**); and, (b) lewis acid-based ring opening of the oxazoline ring of the bicyclic lactams, followed by a 6-endo-trig ring closure to generate fused pyrroloquinolines (from **A** and **A’**). The nitroaryl lactams were further reduced to generate more lipophilic amines. Upon testing this focused library for their anti-malarial activity, dinitro/diamine substituted bicyclic lactams were found most effective growth inhibitory compound against both drug-resistant clone Dd2 and drug-sensitive clones HB3 and 3D7 of *P. falciparum*.

## Methods

### Chemical synthesis

Chiral bicyclic lactams **A** and **A**’ were synthesized from (**R**)-phenylalaninol and levulinic acid and 3-(2-nitrophenyl) levulinic acid. **A** was transformed to **B**_**1**_, **B**_**2**_, **C** and **F** by substituting with various equivalents of *o*-nitrobenzylbromide at the carbon α to the amide functionality or by treating **A** with TiCl_4_*via* a known procedure in the literature [11]. **G** was synthesized in a similar manner as **F**.

The bicyclic lactam **A** was synthesized by condensation of **R**-phenylalaninol with levulinic acid in presence of para-toluenesulfonic acid (PTSA) under toluene reflux with excellent yield (84%) and high diastreoselectivity (98%). Lactam **A** was alkylated with *o*-nitrobenzyl bromide via enolate-mediated alkylation. A typical procedure involved enolizing a tetrahydrofuran (THF) solution of **A** at −78°C with lithium hexamethyl disilyl amide (LHMDS) (2 eq.), followed by addition of *o*-nitrobenzylbromide (1 eq.). The reaction was warmed to room temperature (RT) and was monitored *via* liquid chromatography mass spectroscopy (LCMS). Once it indicated the completion of mono-alkylation (which is after ~12 hour) half of the reaction mixture was syringed to a different flask, then quenched and purified to generate two monobenzylated diastereomers **B1** and **B2**. The remaining reaction mixture was further cooled to −78°C and then treated with LHMDS (2 eq.), and stirred for about 30 minutes, followed by the addition of an equivalent of *o*-nitrobenzylbromide. LCMS monitoring indicated completion of the reaction by next 16 hours, after which it was quenched and purified to obtain the dibenzylated bicyclic lactam **C**. Facile hydrogenation with 10% w/w Pd-C and H_2_ (at atm pressure), of the nitrobicyclic lactams **B1**/**B2** and **C** generated the desired amines **E1**/**E2** and **D**.

In an effort to synthesize compound **A**’, 3-(2-nitrophenyl) levulinic acid (12 g, 50.6 mmol), (**R)**-phenyl alaninol (7.3 g, 52 mmol) and PTSA (12 mg, cat) were dissolved in toluene (120 mL and the flask (equipped with a Dean-Stark trap) was heated to reflux. After 16 hours the solution was cooled, washed with saturated aqueous NaHCO_3_, dried, and concentrated. The final compound was purified by flash column chromatography on silica gel as stationary phase and Hexane/EtOAc, 1:1 as the eluent to afford the desired compound, which was then triturated with diethyl ether to afford the bicyclic lactam intermediate **A**’ (7.4 g, 41.5%) as white solid.

The intermediate **A**’ (500 mg, 1.42 mmol) was treated with titanium chloride (TiCl_4_) (0.4 mL, 3.55 mmol) in dichloromethane (DCM) (15 mL) and the resulting reaction mixture was stirred at 20°C. Once thin layer chromatography (TLC) indicated complete consumption of the starting material, the reaction was quenched with saturated aqueous solution of NaHCO_3_ (20 mL) and was filtered. The organic layer was separated and the aqueous layer was extracted further with dichloromethane (DCM). The combined organic layers were dried over sodium sulphate (Na_2_SO_4_) and evaporated to generate compound **G** in 88:12 diastereomeric ratio. The major product was purified by flash column chromatography. Compounds **B1**, **B2**, **C**, **D**, **E1**, **E2**, **F and G** were dissolved in dimethyl sulfoxide (DMSO; 10 mg/mL) (Sigma-Aldrich Chemie GmbH, Buchs, Switzerland) and stored at 4°C for use as anti-malarials.

### Cell culture

*Plasmodium falciparum* clones used in this study 3D7, Dd2 and HB3 [12–14], were cultured in O^+^ human erythrocytes supplemented with RPMI 1640 (Invitrogen, USA) 24 mM sodium bicarbonate (Sigma, USA), 0.1 mM hypoxanthine (Invitrogen, USA), 25 mg/ml gentamicin (Invitrogen, USA) and 0.5% AlbuMax I (Invitrogen, USA), according to methods described earlier [15]. Parasite culture was maintained in mixed gas environment (5% O_2_, 5% CO_2_ and 90% N_2_). Parasites were synchronized by sorbitol treatment at ring stage.

Kidney fibroblast-like cell lines (COS-7 cells) were cultured in Dulbecco’s Modified Eagle’s Medium (Invitrogen, USA) supplemented with 10% foetal bovine serum (Gibco, USA) and penicillin (100 units/ml) and streptomycin (100 mg/ml). All cultures were incubated under standard culture conditions (37°C, 5% CO2).

### Growth inhibition assays

Compounds were tested at different concentrations for inhibition of growth by *P. falciparum* strains 3D7, HB3 and Dd2. Briefly, the parasites were first synchronized by the purification of schizont-stage parasites on a Percoll gradient, followed by two rounds of treatment of the ring-stage parasites with sorbitol. Schizont-stage parasites at an initial parasitaemia of 1% and at 2% haematocrit were incubated with compounds at concentrations ranging from 0.5-50 μM, untreated as control, for one cycle of parasite growth (40 hours post invasion). Following a 40-hour incubation, the whole sample was collected and washed twice with PBS and stained with ethidium bromide (10 μM) for 15 minutes at room temperature in dark. The cells were washed with PBS, and analysed by flow cytometry on FACSCalibur (Becton Dickinson) using CellQuest software [16]. Fluorescence signal (FL-2) was detected with the 590 nm band pass filter using an excitation laser of 488 nm collecting 100,000 cells per sample. Following acquisition, parasitaemia was estimated by detecting newly infected parasites, by determining the proportion of FL-2-positive cells using Cell Quest.

Growth inhibition (% Inhibition) was calculated as follows:


### Statistical analysis

The data for the IC_50_ values and % parasite growth are expressed as the mean ± standard deviation (SD) of three independent experiments done in duplicates. IC_50_ values were calculated using Graph Pad Prism software.

### Progression assays

The effect of compounds showing most potent growth inhibition (compounds **C** and **D**), was tested on progression of *P. falciparum* 3D7 strain at different stages (rings (R), trophozoites (T), schizonts (S)) and release of merozoites from schizonts. Ring-stage parasite culture was diluted to 2% parasitaemia and 2% haematocrit in a complete RPMI medium and treated with compound (50 μM) or solvent as control and incubated further for 12, 26, 48, and 54 hours blood-stage asexual cycle to monitor progression at each stage. Morphological analysis and counting (~1,000 cells/Giemsa-stained slides in duplicate) were done at each of these stages to monitor progression. Giemsa-stained thin blood smears of *P. falciparum* were made at every 12, 26, 48, and 54 hours post invasion and around 3,000 red blood cells (RBCs) were counted by light microscopy. To determine the relative per cent frequency of different stages of parasite (R, T, S), a pie diagram was made using Graph Pad Prism software.

### Cytotoxicity assay with COS-7 cell line

COS-7 cells seeded in 96-well plates at a seeding density of 30,000 cells per well were allowed to adhere overnight at 37°C. Adhered cells were treated with compounds **C** and **D** at 50 μM concentrations for 24 hours. Cytotoxic effect was assessed using *in vitro* toxicology assay kit (Sigma-Aldrich, St Louis, MO, USA), based on cleavage of MTT ((3-[4,5- dimethylthiazol-2-yl]-2,5-diphenyl tetrazolium bromide)) by viable cells into formazan crystals.

### Terminal deoxynucleotidyl transferase (TdT) mediated dUTP nick end labelling (TUNEL) assay

Parasites at late ring/early trophozoite stages were treated with either compound **D** or solvent alone and incubated for 12 hours. The DNA fragmentation in treated and untreated samples was assessed by TUNEL using In Situ Cell Death Detection Kit, TMR Red (Roche Applied Science), as described previously [17]. Briefly, samples were fixed with 4% paraformaldehyde (Sigma-Aldrich, Fluka Chemicals) and 0.0075% glutaraldehyde in PBS for 30 minutes at room temperature, washed with PBS and permeabilized by 0.01% Triton-X 100. Following this, RBCs were incubated with a mix of TdT enzyme and TMR Red labelled dUTP for one hour at 37°C and washed thrice with 1X PBS. The labelled parasites were then observed in Nikon A1 confocal microscope and the percentage of TUNEL positive cells were calculated.

### JC-1 staining for estimation of mitochondrial membrane potential

The cell permeable lipophilic cation probe JC-1 (Molecular Probes, Eugene, OR, USA) exists as monomers in cytoplasm and emits green fluorescence (525 nm). Within the mitochondria, JC-1 forms aggregates emitting red fluorescence (590 nm) [18]. In healthy cells with high mitochondrial transmembrane potential the aggregates show an intense red fluorescence. Cells in which the potential has been lost show a weak red fluorescence and mostly green fluorescence. A decrease in the red to green fluorescence ratio therefore signifies a loss in the mitochondrial transmembrane potential. To estimate any changes in mitochondrial membrane potential, parasites treated with compound **D** or solvent alone were incubated with 5 μM JC-1 for 30 minutes at 37°C, washed twice with PBS and imaged immediately using a Nikon A1 confocal microscope. To avoid bias, images for both the sets were captured using the same laser intensity, voltage and offset values in all the three channels. For each set, the ratio of red to green fluorescence in 100 parasites from randomly selected fields was estimated. For the estimation of fluorescence intensity in each channel, an infected and stained RBC was selected using the Region of Interest (ROI) tool provided in the Nikon NIS Elements AR Analysis V4.13.04 software. From this ROI area, the fluorescence intensity in each channel was noted as given by the software in ROI statistics. These values were used to calculate the ratio of red to green fluorescence in 100 RBCs in those fields for each set of parasite culture.

## Results

### Synthesis of substituted chiral bicyclic lactams and pyrroloisoquinolines as potential anti-malarials

**Figure 1:**
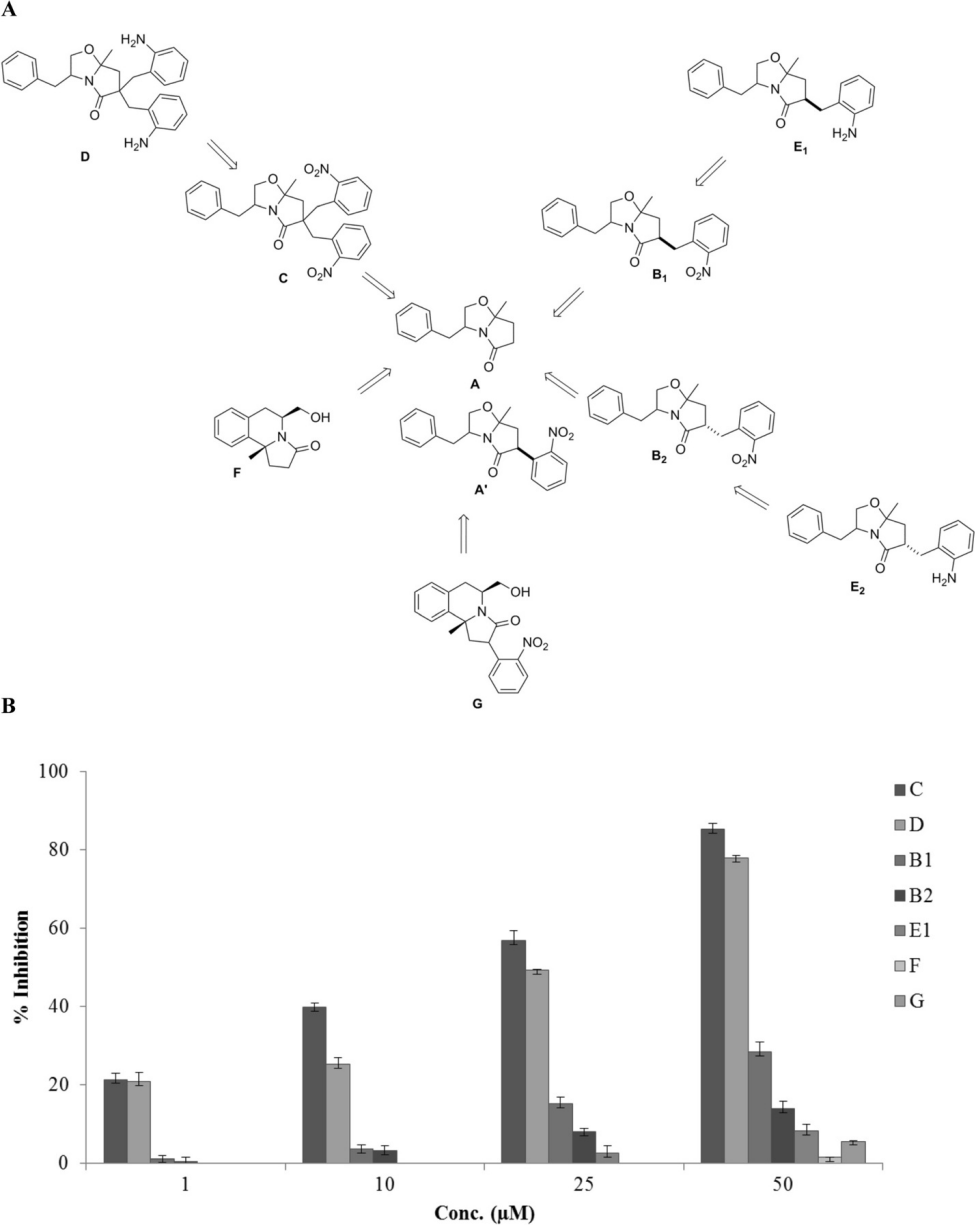
**Overview of the synthesis of substituted chiral bicyclic lactams as potential anti-malarials. (A)** The scheme depicts the synthesis of chiral bicyclic lactams from phenylalaninol/levulinic acid and 3-(2*-*nitrophenyl)levulinic acid. The final compounds were derived either *via* enolization and alkylation with *o*-nitrobenzylbromide and subsequent hydrogenation or by lewis acid based ring opening and subsequent cyclization. **(B)** Screening of synthesized compounds for *P. falciparum* growth-inhibitory activities at four different concentrations of 1 μM, 10 μM, 25 μM, and 50 μM. Bar graph indicates compounds C and D as the most potent inhibitor of *P. falciparum* growth. Three independent assays were performed in duplicates. The error bars show the standard errors of the means.

Substituted chiral bicyclic lactams **B**_**1**_ to **E**_**1**_ were synthesized from **A. A** in turn was synthesized from (**R**)-phenylalaninol and levulinic acid as depicted in the scheme (Figure 1A). The final compounds were derived from **A**, via enolization and alkylation with *o*-nitrobenzylbromide and subsequent hydrogenation.

The diastereoselective alkylation of **A** afforded epimeric *o*-nitrobenzylbicyclic lactams **B**_**1**_ and **B**_**2**_. Their subsequent hydrogenation generated the corresponding amine **E**_**1**_ and **E**_**2**_. In a different reaction, excess of base and prolonged reaction time generated the dialkylated analog **C** that was also hydrogenated to provide the amine **D**. In a separate effort **A** was treated with TiCl_4_ in dichloromethane to undergo the oxazoline ring opening, followed by a 6-endo-trig cyclization to afford **F**. Finally, a novel bicyclic lactam **A**’ was synthesized by condensation of L-phenylalaninol with 2-(2-nitrophenyl)-levulinic acid. In a similar fashion as in **A**’, it was converted to **G**.

### *In vitro*growth inhibitory activity of substituted bicyclic lactams against *Plasmodium falciparum*

Initially the screening of the biological activity of substituted bicyclic lactams was done at different concentrations for inhibition of intraerythrocytic growth by *P. falciparum* strain 3D7. The compounds **C, D, B1, B2, E1, F, and G** (as mentioned in Methods) were tested at different concentrations over one cycle of parasite growth. The untreated parasites served as the control and percentage growth inhibition was measured by flow cytometry. Compound **C** exhibited 39.9 and 85.27% of growth inhibition at 10 μM and 50 μM of concentration, respectively. Compound **D** exhibited 25.24 and 77.84% of growth inhibition at 10 μM and 50 μM of concentration, respectively (Figure 1B, Additional file 1). Concurrently, other compounds exhibited negligible effect (Figure 1B). This indicated that the dinitro-substituted compound **C** and the more lipophilic diamine-substituted compound **D** have more significant effect on growth inhibition compared to the other compounds in the same series (compound **B**_**1** ,_**B**_**2** ,_**E**_**1**_, **F,** and **G**) and are thus the most potent of all the substituted bicyclic lactams tested.

### Half maximal inhibitory concentration (IC_50_) of compound C and compound D

**Figure 2:**
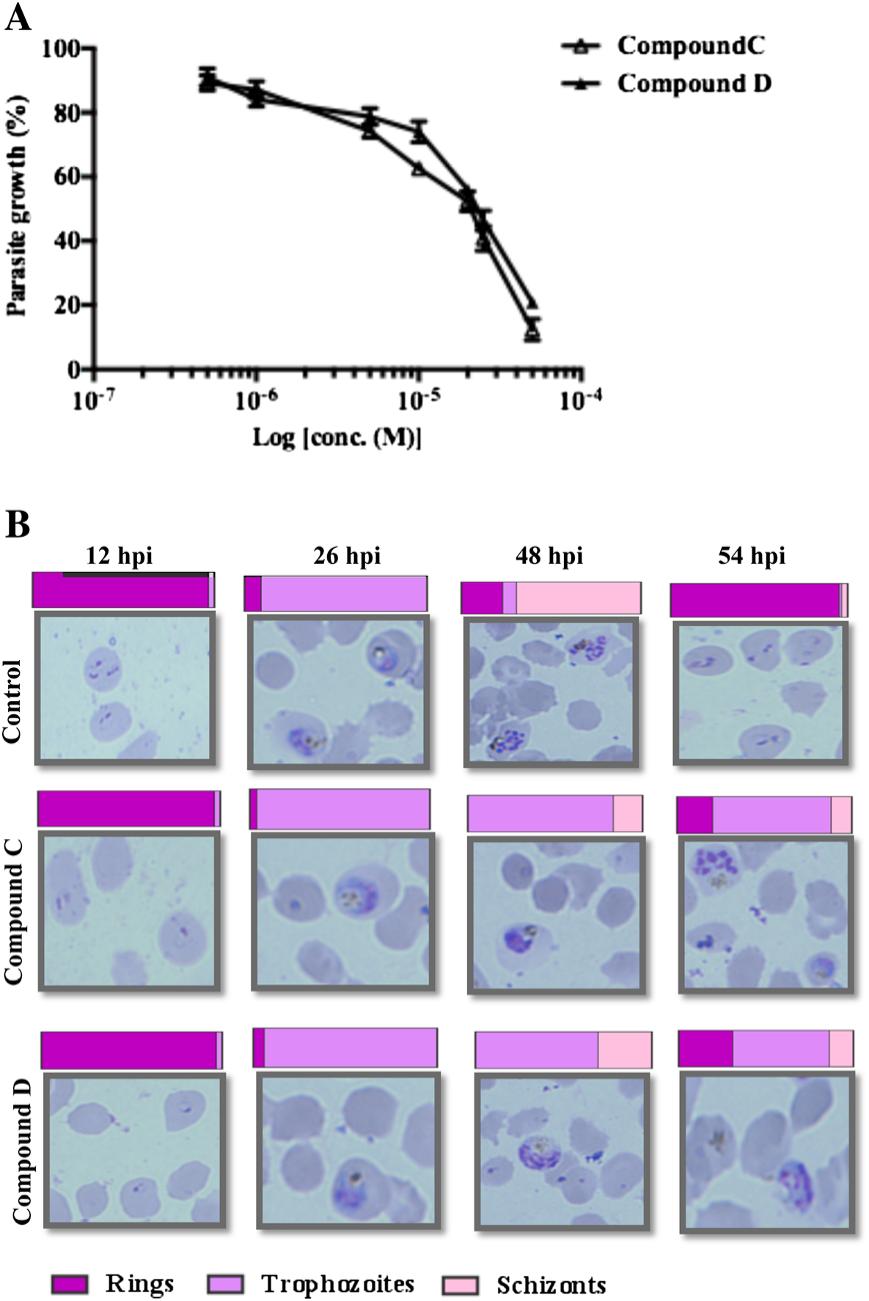
**Effect of compounds C and D on parasite growth. (A)** Percent parasite growth curve showing the effect of compounds C and D on *P. falciparum* growth at different concentrations of 0.5 μM, 1 μM, 5 μM, 10 μM, 20 μM, 25 μM, and 50 μM. Three independent experiments were performed in duplicates. **(B)** Comparison of parasite intra-erythrocytic maturation in presence and absence of compounds C and D. Light microscopy Giemsa-stained images of *P. falciparum-*infected RBCs at 12, 26, 48, and 54 hpi incubated with and without compounds. Bars indicate relative percent frequency of parasite ring, trophozoite and schizont stages in the 12, 26, 48, and 54 hpi. Healthy trophozoites are observed 26 hpi, either in presence or absence of compounds. At 48 hpi, healthy new rings are observed in untreated control but, are >70% reduced in the presence of 50 μM of compound C and compound D with parasite growth arrested as early and late trophozoites.

Compounds that demonstrated the most significant effect on parasite growth were further investigated for the determination of the half maximal inhibitory concentration (IC_50_). Exploration of the half maximal inhibitory concentrations (IC_**50**_) of compound **C** and **D** in dose-dependent manner exhibited the IC_50_ value of 19.23 μM and 23.03 μM, respectively (Figure 2A). The IC_50_ value is based on the values after 40–44 hours of sorbitol synchronized *P. falciparum* 3D7 clones at schizont-stage at different concentrations of 0.5 μM, 1 μM, 5 μM, 10 μM, 20 μM, 25 μM, and 50 μM.

### Stage-specific effect of compounds on *Plasmodium falciparum*progression *in vitro*

Morphological analysis and counting (~3,000 cells/Giemsa-stained slides in duplicate) over the 48-hour blood stage cycle revealed healthy trophozoites 26 hours post-invasion (hpi) either in presence or absence of compounds (Figure 2B). At ~48 hpi healthy schizonts and new rings were observed in solvent control but were >70% reduced in the presence of either compound **C** or, compound **D** (Figure 2B). Parasites remained stalled at early to late trophozoites on treatment with compound **C**, **D** even at ~54 hpi.

### Growth inhibition activity of compounds across three different strains of *Plasmodium falciparum*

**Figure 3:**
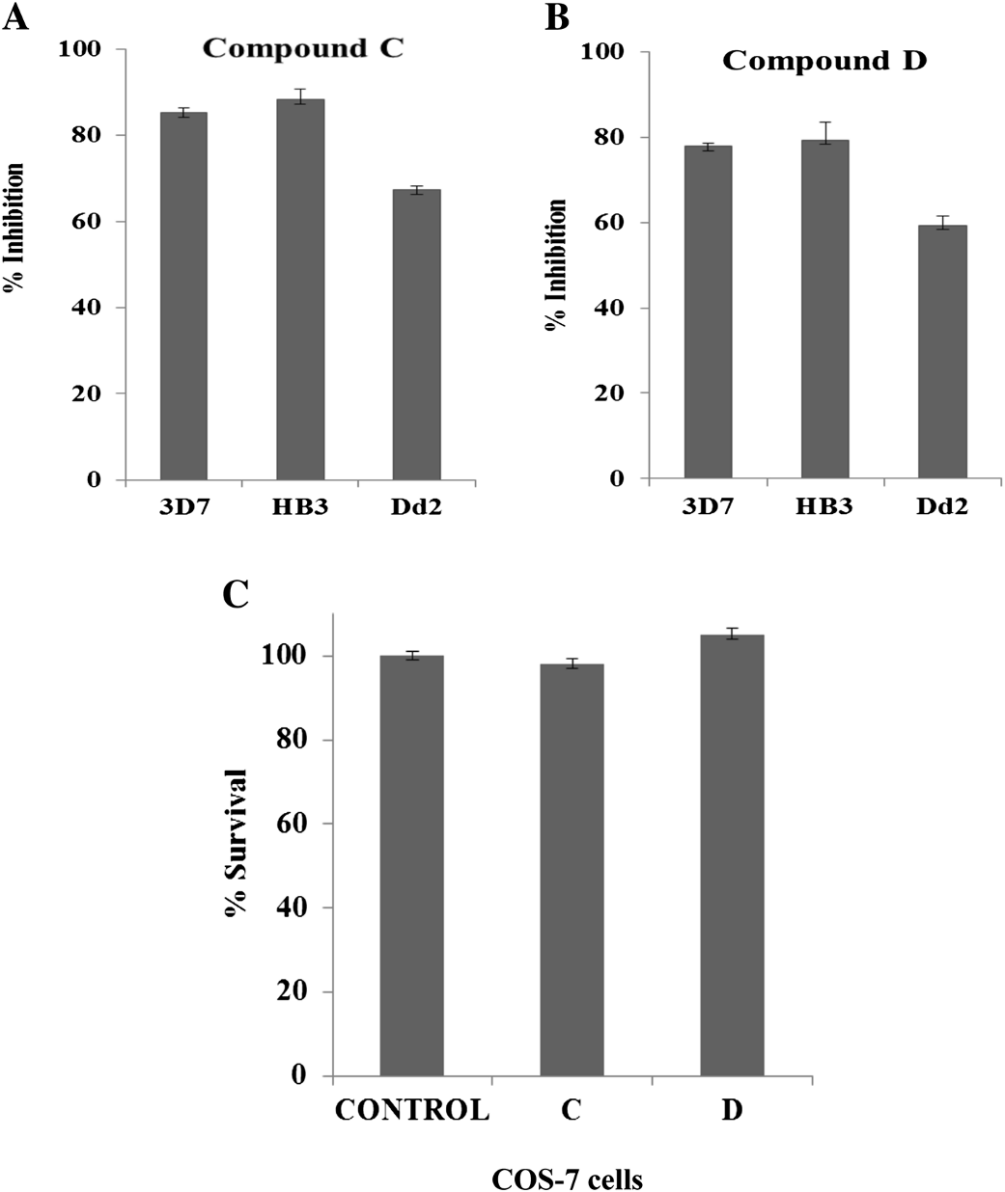
**Growth-inhibitory activities of compounds across different** ***Plasmodium falciparum*** **strains and on viability of COS-7 cells. (A)** Bar graph showing the growth-inhibitory effect of dintro-substituted compound C at 50 μM concentration on sorbitol synchronized *P. falciparum* drug-sensitive clones 3D7, HB3 and drug-resistant clones Dd2. **(B)** Bar graph showing the growth inhibitory effect of diamine-substituted compound **D** at 50 μM concentration on sorbitol synchronized *P. falciparum* drug-sensitive clones 3D7, HB3 and drug-resistant clones Dd2. Three independent assays were performed in duplicates. The error bars show the standard errors of the means. **(C)** Bar graph showing percentage survival of COS-7 cells after 24 hours of treatment with compounds C and D. Three independent assays were performed in duplicates. The error bars show the standard errors of the means.

Aforementioned results showed compounds **C** and **D** to be the most effective for growth inhibition in *P. falciparum* drug-sensitive strain 3D7. In order to further evaluate the potential of compounds **C** and **D**, the growth inhibitory activity of these compounds was assessed against drug-resistant *P. falciparum* clone Dd2 and drug-sensitive *P. falciparum* clone HB3 along with 3D7, over one cycle at 10 μM and 50 μM of concentrations (Figure 3A,B). Compound **C** exhibited the 48.6 and 39.9% of inhibition in drug-sensitive clones HB3 and 3D7, respectively, whereas 34.24% in drug-resistant clone Dd2 at 10 μM of concentration; also at 50 μM of concentration, it showed 85.27 and 88.35% of inhibition in drug-sensitive clones 3D7 and HB3, respectively, whereas 67.27% in drug-resistant clone Dd2. Compound **D** exhibited the 77.84 and 79.34% of inhibition in drug-sensitive clones 3D7 and HB3, respectively, whereas 59.3% in drug-resistant clone Dd2 at 50 μM of concentration (Figure 3A,B). It is evident from these data that the compounds show efficacy against both drug-sensitive as well as drug-resistant clones of *P. falciparum.*

### Effect of compounds on mammalian cell viability

Compounds **C** and **D**, having significant effect on growth of malaria parasite, were further assessed for cytotoxic effects on COS-7 mammalian cell line. The degree of cytotoxicity caused was measured spectrophotometrically, based on the protocol described previously by Mossmann [19]. No significant cytotoxicity was observed at concentration as high as 50 μM for both compounds **C** and **D** treated cells.

### Substituted bicyclic lactam compound D reduces parasite-mitochondrial membrane potential

**Figure 4:**
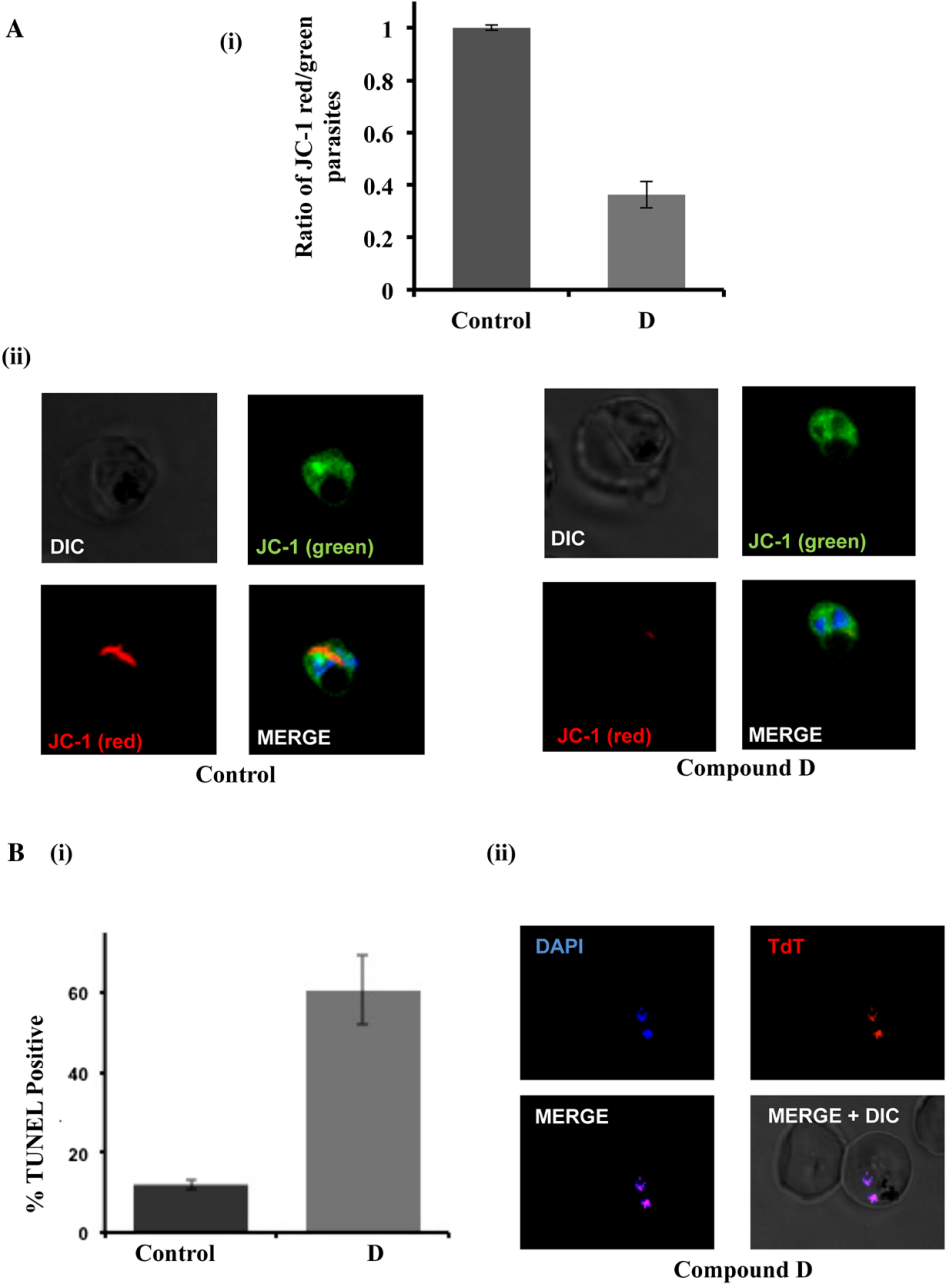
**Loss of mitochondrial membrane potential and apoptosis like cell-death in compound D-treated** ***Plasmodium falciparum*** **parasites. (A)** (i) Bar graph showing reduction in ratio of JC-1 (red)/JC-1 (green) in parasite population after treatment with compound D. Two independent experiments were performed in duplicates. The error bars show the standard errors of the means. (ii) Fluorescent images of JC-1-stained parasites showing aggregated JC-1 (red) in the mitochondria and monomeric JC-1 (green) in the cytoplasm after treatment with solvent alone or compound D. Parasite nuclei were stained with DAPI (blue). **(B)** (i) Bar graph showing percentage of TUNEL-positive parasites after treatment with compound D as compared to solvent alone (control). Two independent assays were performed in duplicates. The error bars show the standard errors of the means. (ii) Fluorescent images of parasites stained with TMR Red (TUNEL staining) showing DNA fragmentation after treatment with compound D.

The treated parasites were further examined for features associated with cell death, alteration in mitochondrial membrane potential and apoptosis. Ratio of JC-1 (red)/JC-1 (green) was calculated to assess the loss of mitochondrial membrane potential. The JC-1 staining was observed as strong red mitochondrial fluorescence and negligible diffused green fluorescence (JC-1 monomer) across the erythrocyte and parasite’s cytoplasm in solvent-treated parasites, characteristic of a functional mitochondria as (Figure 4A (i), (ii)). On the contrary, diffused green fluorescence (JC-1 monomer) was observed in ~60% of compound **D**-treated parasites, suggesting loss of mitochondrial membrane potential (Figure 4A (i), (ii)).

### Compound D-induced apoptosis as detected via TUNEL assay

Next, DNA fragmentation was assessed in treated and untreated samples by TUNEL using In Situ Cell Death Detection Kit, TMR Red (Roche Applied Science). The labelled parasites were observed in Nikon A1 confocal microscope and the percentage of TUNEL-positive cells were calculated. The percentage of TUNEL-positive parasites after treatment with compound **D** increased by 60% compared to solvent control (Figure 4B (i)). Also the fluorescent image of parasites treated with compound **D**, stained with TMR Red (TUNEL staining) showed DNA fragmentation (Figure 4B (ii)).

## Discussion

Owning to immediate need of development of anti-malarials, a new class of substituted bicyclic lactams was developed and evaluated for their anti-malarial activity against the asexual erythrocytic stage of *P. falciparum.* Lactams are reported as a large class of antibiotics characterized by the presence of the azetidin-2-one/oxazol-2-one rings, which is the core of their biological activities. They are further differentiated by side chains, unsaturations, heteroatoms, and, in many cases, by the presence of another five- or six-membered rings [20, 21]. Biologically active lactams range from β-lactams, used in diverse therapeutic areas *viz* serine-dependent enzyme inhibitors, matrix-metalloprotease inhibitors, and even apoptosis inductors [22, 23], the inhibition of HIV-1 protease, antitumor activity, anti-malarial activity, and cholesterol absorption inhibition [24–30], peptides, bicyclic lactams (as potential anticancer and anti-malarials) etc. This study, reports the genesis (from DOS) of substituted bicyclic lactams and their cyotoxic effect on malaria parasite growth, with the objective to contribute new anti-malarial agents against *P. falciparum*. It is not the first time DOS has been used to generate efficacious small molecules against malaria. Schreiber *et al.* has relentlessly researched on various kinds of molecules ranging from small molecules to macrocycles evolved from DOS towards generating a therapeutic compound against malaria. NITD-609, Novartis’ recent molecule in phase 2 clinical trials for malaria is also the result of a DOS inspired effort. Additionally Brown *et al*. also demonstrated the prowess of DOS in generating antimalarial compounds [31–35]. A DOS library was generated from **A** and **A’** by direct substitution with *o*-nitrobenzylbromide at the carbon α to the amide functionality and also converting **A’** into fused pyrroloquinolines. A rational chemistry-based approach is undertaken to design molecules modifying the compounds for less toxicity, bioavailability, more stability, solubility, and other attractive features [36]. Based on preliminary screening results, the dinitro-substituted bicyclic lactam has emerged as the most active compound in the series. The nitro group in compound **C** was consequently reduced to amine (compound **D**) as amines are more soluble due to their hydrophilic nature and hence are more bio-available compared to their nitro counterpart. The nitroaryl lactams (compound **C**) and the further reduced, more lipophilic amine forms (compound **D**) demonstrated exceptional activity, showing >85% inhibition at 50 μM concentration across different drug-sensitive and drug-resistant strains of *P. falciparum* (Figure 3A,B). It is noteworthy that the introduction of modification attributed not much change in antiparasitic activity as both compounds showed significant effect on the parasite growth and exerted no cytotoxic effect on mammalian cell line (Figure 3A,B,C). Amines, being more soluble owing to their hydrophilic nature, are more bio-available as compared to their nitro counterpart, which will benefit further *in vivo* antiplasmodial evaluation of the compound. Also the death of parasite following *in vitro* exposure to compound **D** was investigated using biochemical and morphological approaches. Diffused green fluorescence of JC-1 monomer observed in compound **D**-treated parasites suggested a loss of mitochondrial functionality and thus mitochondrial membrane potential. TUNNEL assay further revealed the presence of apoptotic cells in compound **D**-treated parasites. This study thus, establishes a strong demonstration of the superior efficacy and cytotoxicity of substituted bicyclic lactams against a broad range *of P. falciparum* strains. These findings advocate a new class of compounds, which could be considered as potential anti-malarials and inclusion of these compounds in combination-based therapy seems promising. The *in vivo* assessment of the antiparasitic potential of these bicyclic lactams could be beneficial to achieve success in development of novel anti-malarials and in eradication of this devastating disease. Last but not the least the initial lead compound obtained *via* our DOS strategy can be further elaborated and improved into a better drug candidate in the future. There are quite a few sites of diversity present in the molecules for example the aromatic ring of the benzylic moiety, the oxazoline side ring which can be changed to thiazoline side chain, the subsequent reduction of the nitro group followed by diverse array of acid-amine coupling or C-N bond formation reactions with aliphatic, aromatic and heteroaromatic halides and finally, further substituting the position α- to the amide carbonyl either by alkylation or by acylation will provide opportunities to improve the efficacy of this initial lead compound.

## Electronic supplementary material

Additional file 1: **Growth inhibition activity of substituted bicyclic lactams. Growth-inhibitory activities of compounds C, D, B**_**1**_ **, B**_**2**_ **, E**_**1**_ **, F, and G.** Compounds were assayed at two different concentrations of 10 μM and 50 μM in sorbitol synchronized *P. falciparum* 3D7 clones. Three independent assays were performed in duplicate. ^§^SEM is standard error of means. (TIFF 284 KB)
