# Supplementary figures and images for: Diversity-oriented synthesis and activity evaluation of substituted bicyclic lactams as anti-malarial against Plasmodium falciparum
Source: Malar J. 2014 Nov 28;13:467. doi: 10.1186/1475-2875-13-467 (PMC4289231; doi:10.1186/1475-2875-13-467)

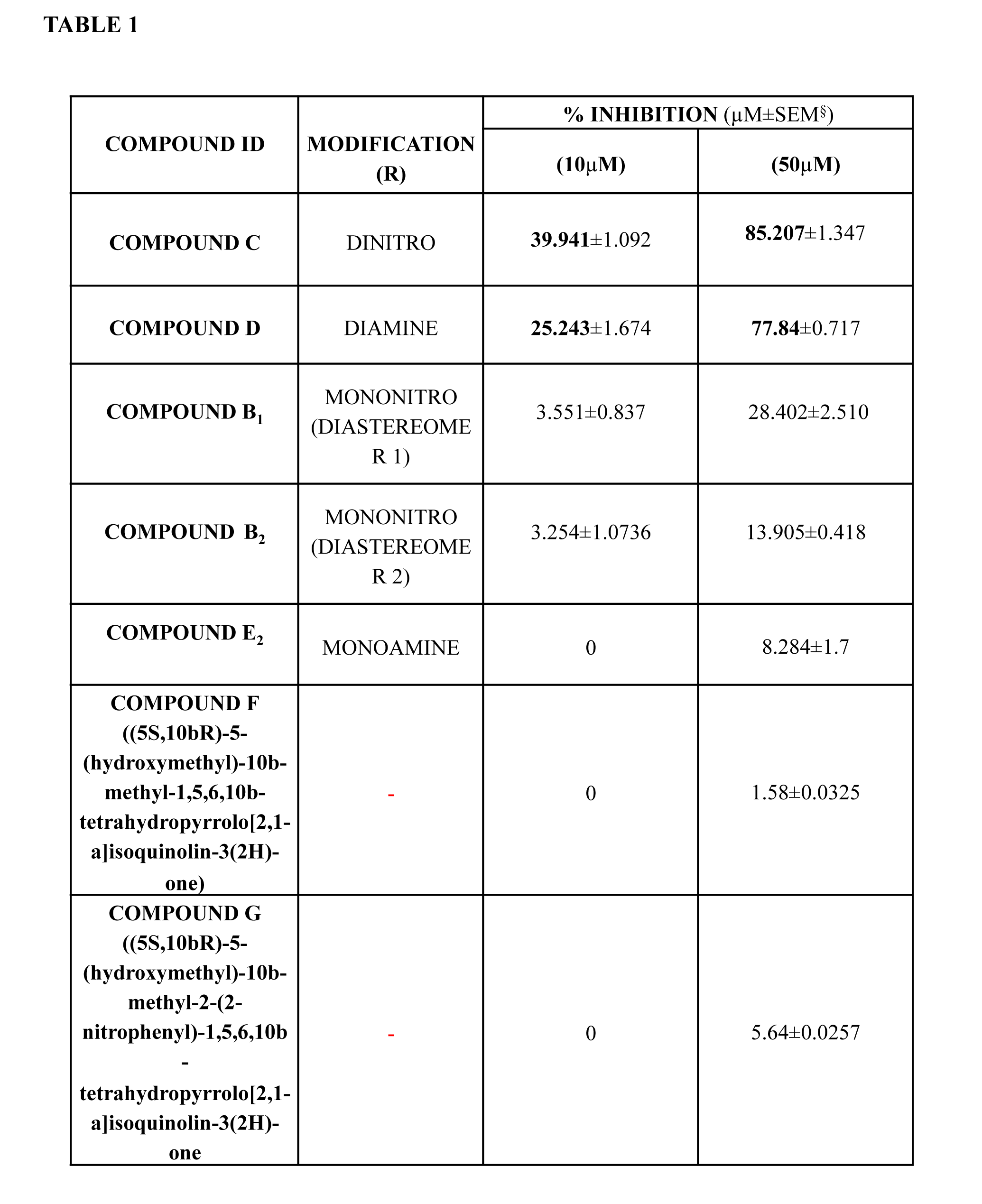

Supplement: Supplementary file 1 — Additional file 1: Growth inhibition activity of substituted bicyclic lactams. Growth-inhibitory activities of compounds C, D, B 1 , B 2 , E 1 , F, and G. Compounds were assayed at two different concentrations of 10 μM and 50 μM in sorbitol synchronized P. falciparum 3D7 clones. Three independent assays were performed in duplicate. §SEM is standard error of means. (TIFF 284 KB) [file 12936_2014_3651_MOESM1_ESM.tiff]
